# Flecainide Toxicity in a Patient with a Functioning Pacemaker

**DOI:** 10.19102/icrm.2024.15075

**Published:** 2024-07-15

**Authors:** Sneha Chebrolu, Jonathan Mayl, Prashant Bhave

**Affiliations:** 1 Department of Internal Medicine, Wake Forest Baptist Health, Winston-Salem, NC, USA; 2 Department of Cardiology, Wake Forest Baptist Health, Winston-Salem, NC, USA

**Keywords:** Anti-arrhythmic, ECG, flecainide toxicity, pacemaker, use dependence, ventricular pacing

## Abstract

Flecainide is a class Ic anti-arrhythmic that demonstrates use dependence, meaning the medication has an increased effect on the myocardium at high heart rates. Flecainide toxicity can be identified by wide QRS complexes on an electrocardiogram (ECG). We discuss a case of a 75-year-old patient with a pacemaker who presented with concern for flecainide toxicity. The patient had several risk factors known to increase the likelihood for toxicity, including structural heart disease and acute kidney injury. The initial ECG showed tachycardia with wide QRS complexes. The patient had a pacemaker set in a tracking mode (DDD) that resulted in rapid ventricular pacing with failure to mode switch. However, with modification to the VVI mode, the patient experienced tachycardia resolution with an improvement in QRS complexes. This case emphasizes the use dependence of flecainide and illustrates the utility of pacing mode in the management of flecainide toxicity in patients with pacemakers.

## Introduction

Flecainide is a commonly used medication in the treatment of atrial fibrillation that demonstrates use-dependence effects. There is a limited description of flecainide toxicity in patients with pacemakers in the literature. We present a case of flecainide toxicity in a patient with an inappropriately tracked atrial flutter with rapid pacing that improved with reprogramming of the pacing mode. This case shows that modification of pacemaker settings can be used in the management of flecainide toxicity.

## Case presentation

We describe a case of a 75-year-old man with atrial fibrillation and sick sinus syndrome with dual-chamber pacemaker placement in 2020 who presented from an outside hospital with shortness of breath, palpitations, and generalized weakness. He was initially hypotensive with a blood pressure of 94/75 mmHg but responsive to fluids and tachycardic at 118 bpm with concern for pacemaker capture failure. Before being transferred, he was noted to have an increased ventricular pacing threshold of 3.0 V at 0.4 ms. During his previous device interrogation, his ventricular pacing threshold was lower (2.0 V at 0.4 ms), with 61% ventricular pacing. Upon arrival at our facility, his device was found to be functioning normally upon interrogation, with an increase in output to 7.0 V at 0.4 ms.

He had a normal stress test several months after his pacemaker implantation prior to flecainide initiation. Notably, flecainide was previously held due to a mild reduction in his ejection fraction (EF) to 40%–45% on a transthoracic echocardiogram (TTE) 1 year before presentation. He then experienced an improvement in his EF to >55% several months later. He was noted to have a high burden of atrial fibrillation and restarted on flecainide 100 mg twice a day. It is uncertain whether an electrocardiogram (ECG) was checked in preparation for resumption to ensure no QRS prolongation. He was seen by his outpatient cardiologist a few days before his presentation and found to be back in atrial fibrillation. Consequently, his flecainide dose was increased to 200 mg twice a day, which exceeds the typical maximum dose. Of note, he was also treated with metoprolol tartrate and apixaban for anticoagulation.

**Figure 1:**
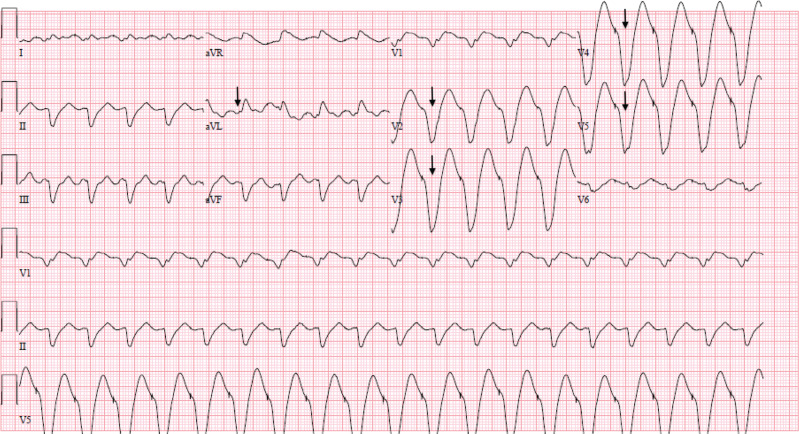
Initial electrocardiogram demonstrating a slow atrial flutter with a ventricular paced rhythm tracking 2:1 (arrow in aVL notating p-wave) and profoundly wide QRS characteristic of flecainide toxicity (arrows in precordial leads).

**Figure 2:**
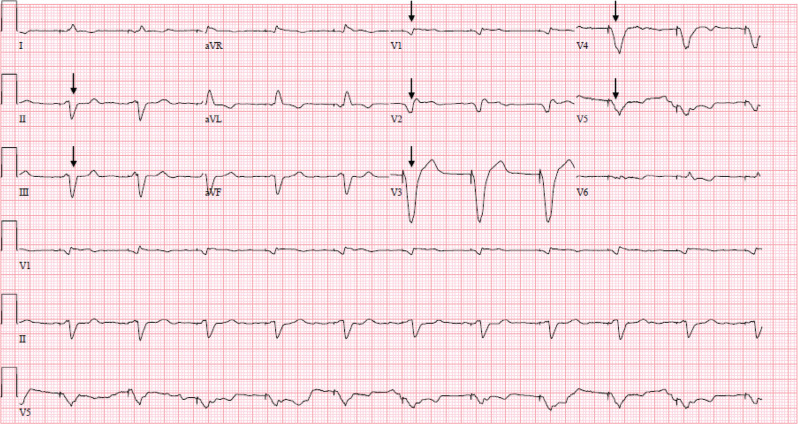
Subsequent electrocardiogram demonstrating ventricular paced rhythm after pacemaker reprogrammed to VVI with a base rate of 65 bpm. There is a dramatic narrowing of the QRS (arrows) with the change in rate.

**Figure 3:**
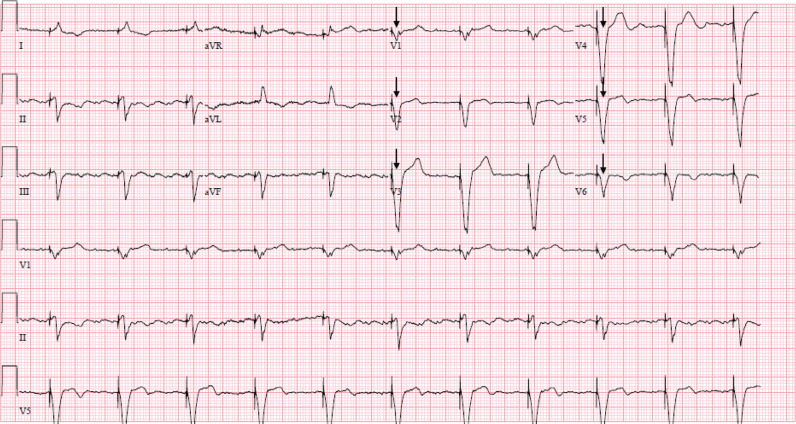
Electrocardiogram recorded on day 7 of hospitalization demonstrating atrial flutter with a ventricular paced rhythm. The QRS width has improved (arrows).

His initial ECG on presentation demonstrated ventricular pacing at 120 bpm and extreme widening of the QRS complex to 280 ms **(Figure 1)**. His last ECG obtained the month prior to admission showed a QRS of 196 ms and a corrected QT of 558 ms. Initial admission laboratory results included the following: sodium (Na), 131 mmol/L; potassium (K), 5.2 mmol/L; magnesium (Mg) 1.5 mg/dL; creatinine (Cr), 1.2 mg/dL (baseline, 0.9 mg/dL); and lactic acid, 2.3 mmol/L. Flecainide was held on admission given the concern for toxicity with widening of the QRS complex. His device interrogation revealed atrial flutter, which was being tracked 2:1 by the pacemaker. Though he was prescribed apixaban, he was not compliant; therefore, cardioversion was not immediately performed on presentation. The device mode was changed from DDD to VVI and set to a base rate of 65 bpm to maintain an adequate heart rate without potentiating flecainide effects. He subsequently experienced narrowing of the QRS complex to 200 ms **(Figure 2)** and 160 ms 7 days later **(Figure 3)**. The device-pacing threshold was not assessed after the QRS had narrowed.

TTE performed the day prior to transfer demonstrated an EF of 40%–45%. However, at our facility, he was found to have further reduced systolic function, with an EF of 20%–25% and severe hypokinesis of the mid- to apical anterior and anterolateral walls. He underwent coronary angiography, which showed multivessel coronary artery disease. His device was interrogated again and showed persistent atrial flutter. He underwent successful cardioversion with a concurrent transesophageal echocardiogram. After sufficient washout of flecainide, he was started on amiodarone. He had a repeat TTE 1 month after discharge that confirmed an improvement in EF to 35%–40%.

## Discussion

Flecainide is a class Ic anti-arrhythmic used in the management of atrial fibrillation. Flecainide inactivates Na channels to slow depolarization and can lead to QRS prolongation. More specifically, flecainide has increased affinity to Na channels in the open state. This results in use dependence, meaning that flecainide has a greater effect on the myocardium at higher heart rates.^1^ Previous reports of flecainide toxicity have demonstrated ECG findings, including right and left bundle branch block and a wide QRS complex.^2,3^ The patient presented with tachycardia and was noted to have a significantly widened QRS. This raised concern for flecainide toxicity as the medication dose had been recently increased. While flecainide can affect both the conduction system and myocardium, the impact on cardiac muscle was the driving factor for the widened QRS in the context of a ventricular paced rhythm. He was also found to have a chronically elevated ventricular pacing threshold that was further increased by flecainide effects.

In this case, the development of flecainide toxicity is probably multifactorial. Flecainide use is contraindicated in patients with structural heart disease and coronary artery disease as it has been shown to increase mortality.^4^ The patient was found to have a reduced EF compared to a prior TTE, which could have contributed to the toxic effects. The patient’s reduced EF may have been a result of tachycardia cardiomyopathy in the setting of persistent atrial fibrillation and flutter, right ventricular pacing–induced cardiomyopathy given his high levels of ventricular pacing, and the negative inotropic effect of flecainide. Mild acute kidney injury may have also contributed as flecainide is partially cleared renally. Given the high clinical suspicion for flecainide toxicity, a serum flecainide level was not obtained before intervention. The case was further complicated by rapid ventricular pacing, which was related to the pacemaker being set in a tracking mode (DDD) in the context of atrial flutter. Failure to mode switch was due to every other flutter wave falling into the post-ventricular atrial blanking period.

With reprogramming to the VVI mode at 65 bpm, the paced QRS complex narrowed in duration **(Figures 2 and 3)**. The patient could also have been switched to a non-tracking mode (DDI). In the context of atrial flutter, the atrial rate is above the base rate; therefore, DDI would perform similarly to VVI. Further, flecainide affects both atrial and ventricular muscle by slowing conduction velocity; as such, the atrial rate was slower than normal atrial flutter in this case. The patient may have benefitted from immediate cardioversion if anticoagulation compliance was certain by slowing the QRS. However, changing to VVI accomplished a similar result with regard to the QRS. There is a reported case of flecainide toxicity in a patient with a pacemaker, but, in that case, the toxicity caused pacemaker failure.^5^ Typical management of flecainide toxicity includes the use of sodium bicarbonate; activated charcoal; and, in some instances, intravenous fat emulsion and extracorporeal membrane oxygenation to counter the effects of flecainide.^2,6^ It is important to note that switching the pacemaker mode does not decrease flecainide levels but rather minimizes the use-dependence effects of the medication. This case presents the utility of pacemaker mode switching into a non-tracking mode to address the use-dependence effects of flecainide.

## Conclusion

This case demonstrates the use-dependence effect of flecainide; the ECG changes improved by reducing the heart rate. In this scenario, a reduction in heart rate was achieved by reprogramming the pacemaker to change the pacing mode.
